# Enabling Intelligent IoTs for Histopathology Image Analysis Using Convolutional Neural Networks

**DOI:** 10.3390/mi13081364

**Published:** 2022-08-22

**Authors:** Mohammed H. Alali, Arman Roohi, Shaahin Angizi, Jitender S. Deogun

**Affiliations:** 1 School of Computing, University of Nebraska-Lincoln, Lincoln, NE 68588, USA; 2 Department of Computer Science, Prince Sattam Bin Abdulaziz University, Al-Kharj 16273, Saudi Arabia; 3 Department of Electrical and Computer Engineering, New Jersey Institute of Technology, Newark, NJ 07102, USA

**Keywords:** quantization, convolutional neural network, histopathology image analysis, low power classifier

## Abstract

Medical imaging is an essential data source that has been leveraged worldwide in healthcare systems. In pathology, histopathology images are used for cancer diagnosis, whereas these images are very complex and their analyses by pathologists require large amounts of time and effort. On the other hand, although convolutional neural networks (CNNs) have produced near-human results in image processing tasks, their processing time is becoming longer and they need higher computational power. In this paper, we implement a quantized ResNet model on two histopathology image datasets to optimize the inference power consumption. We analyze classification accuracy, energy estimation, and hardware utilization metrics to evaluate our method. First, the original RGB-colored images are utilized for the training phase, and then compression methods such as channel reduction and sparsity are applied. Our results show an accuracy increase of 6% from RGB on 32-bit (baseline) to the optimized representation of sparsity on RGB with a lower bit-width, i.e., <8:8>. For energy estimation on the used CNN model, we found that the energy used in RGB color mode with 32-bit is considerably higher than the other lower bit-width and compressed color modes. Moreover, we show that lower bit-width implementations yield higher resource utilization and a lower memory bottleneck ratio. This work is suitable for inference on energy-limited devices, which are increasingly being used in the Internet of Things (IoT) systems that facilitate healthcare systems.

## 1. Introduction

Medical imaging is an essential data source is used worldwide for disease analysis, diagnosis, and prognosis. Many types of medical images span the wide field of medicine. Radiology is the branch of medicine that uses radiation-based images such as bone X-ray, brain MRIs, cardiac ultrasound, and liver CT to diagnose diseases and guide treatment. Pathology, on the other hand, focuses on studying the causes and effects of diseases using different kinds of images, such as histopathology, cytopathology, and dermatopathology images. Histopathology is the study of diseases using tissue changes from histopathology images.

Medical imaging is characterized by a variety of shapes and sizes. On the one hand, in the medical field, machine learning (ML) and deep learning (DL) have led to significant advancements in image processing. One of the main machine learning methods in image processing is convolutional neural networks (CNNs), which have produced near-human results in image processing. However, most state-of-the-art CNNs are very deep and complex and require numerous parameters to be trained properly. Thus, training these CNNs takes quite some time, such as weeks or months. Many well-known CNN models have been used extensively in computational pathology, such as ResNet [1]. On the other hand, power-limited devices are widely used in medical applications, but they cannot handle deep and complex CNNs. Hence, there is a need for software and hardware optimization techniques to deploy CNNs on low-powered devices. One powerful form of optimization is quantization. In quantization, lower bit-width parameters are used instead of the 32-bit full bit-width parameters.

In this paper, we develop and implement a pipeline-based approach for the EWGS model [2] that uses optimization techniques to perform machine learning for the quantization of histopathology images. We choose quantization as an acceleration technique to control bit-precision, such as the width of the bits (bit-width) employed in the development of CNNs. We plan to design an acceleration method that produces classification accuracy comparable to the full-precision standard method. We aim to perform inference on IoT devices, while training is generally done on GPUs. We use scalar quantization that maps a large set of numbers to a smaller set [3].

## 2. Background

### 2.1. Related Works in Medical Imaging

**Figure 1:**
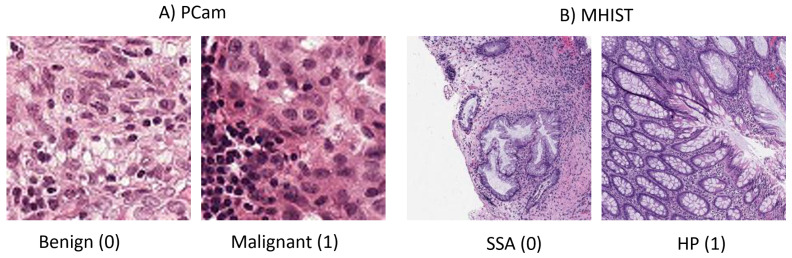
Two sample images from each of the two datasets. (**A**) Images from PCam. (**B**) Images from MHIST, with one for each class.

Considerable medical disciplines utilize imaging for the diagnosis of various diseases, such as radiology, pathology, and dermatology. A number of recent review papers have surveyed the use of deep learning (DL) in medical image classification, object detection, segmentation, and registration [4,5,6,7,8,9,10]. Another review of commonly used CNNs in medical imaging, such as ResNet and GoogleNet, is presented by [11]. Varoquaux and Cheplygina 2022 discuss the challenges and pitfalls that impede the progress of ML applications in medical images, such as data limitations. They also present some recommendations for enhancing ML application research, such as using higher standard benchmarking for evaluation [12]. A brief and practical tutorial for writing the first DL application in medical imaging is provided by [13]. Another review of recent advanced and common medical imaging modalities is given by [14]. The paper also discusses common problems such as class imbalance in medical datasets and possible solutions, such as evaluating the imbalanced dataset on different loss functions. There is an inherent problem in most medical images where the pattern difference is difficult to notice by the human eye. Refer to Figure 1 to see that the benign and malignant images could appear similar. This issue is caused by the noisy labeling of images and is investigated by [15]. The authors proposed a novel strategy based on co-training using global and local representations that is applied to the PCam dataset.

### 2.2. Related Works in Computational Pathology

Computational pathology is the study of pathology images using computational tools, and, herein, we specifically focus on histopathology images, which are histology images that pathologists use to diagnose diseases or cases. The research area of computational pathology recently developed primarily due to advances in image processing, especially by using CNNs. The main medium used in computational pathology is whole slide images (WSI), which are very large images that may reach 10 k × 10 k pixels in size. Researchers have studied numerous computational pathology aspects, applied to various cancer types [16,17,18]. Since directly processing WSI is complicated and challenging, a novel and compressed representation of WSI is proposed by [19]. This representation is based on the cellular information in the WSI and can improve the prediction performance by a maximum of 26% compared to the original WSI. The work by [20] proposed using color adjustment techniques for classifying histopathology images, and they focused on the PCam dataset. They used an ensemble of CNN models to assess the type and found that accuracy results obtained using a combination of color adjustments are better than those with the original RGB color.

### 2.3. Hardware-Friendly Neural Networks

Machine learning is widely applied in diverse fields in which small and power-limited devices are increasingly utilized, resulting in a high demand for them. These devices are used in Internet of Things (IoT) systems. Hence, to run machine learning, specifically CNNs, on low-energy devices, we need to introduce optimization and acceleration techniques in order to obtain reliable accuracy results. Quantization is an optimization technique that reduces the precision of floating-point numbers for calculations. The goal of quantization is to speed up processing, reduce memory usage, and lower power consumption.

There are numerous research papers that apply quantization to histopathology images. A version of MobileNetV2 was designed, quantized, and tested on PCam in [21]. The authors observed that the quantized model produced better accuracy results than the full-precision model. Quantization on CNNs applied to portable devices is discussed in [22,23,24,25], where the authors evaluated inference results on medical images using a quantized CNN and exhibited that the inference time is reduced by 97%. The objective of quantizing a neural network is to decrease the bit-width of the network parameters: weights and/or activation. Quantization is useful in reducing the number of bytes required for storing a model and reducing the computational cost of a convolutional neural network (CNN). It can therefore be used in limited hardware devices. It is worth noting that quantization is mainly used for optimizing inference rather than for training.

## 3. Dataset

Many datasets of histopathology images are available for research use. In this work, we select datasets of smaller images that are more suitable for the application of low-power IoT devices, including the PatchCamelyon (PCam) [26] and the Minimalist Histopathology (MHIST) [27] datasets. Figure 1 shows sample images from each dataset.

### 3.1. PCam Dataset

The PatchCamelyon (PCam) dataset [26] that is derived from Camelyon16 is one of the datasets used in this paper. The PCam consists of 327,680 histopathologic color images of 96 × 96 pixels. Each image represents the presence or absence of metastatic tissue, making it a binary classification task. Class 1 represents almost 50% of the images in the dataset. Table 1 shows a breakdown of the number of images in each class and each phase of deep learning.

### 3.2. MHIST Dataset

The second dataset used in this paper is called the Minimalist Histopathology (MHIST) image analysis dataset, which was introduced by [27]. It is also a binary classification dataset, with 3152 images of size 224 × 224 pixels. This dataset consists of colorectal polyp images with two classes: Hyperplastic Polyp (HP) and Sessile Serrated Adenoma (SSA). HP cases are benign and SSA cases are nearly malignant. Table 2 contains a breakdown of the number of images in each class and in each phase of deep learning.

## 4. Proposed Approach

Herein, the two datasets are first loaded, and then preprocessing is applied as described in Section 4.3. The deep learning model is trained on 32-bit precision. The same model is trained on lower bit-widths. We use EWGS, an element-wise gradient scaling method for training a quantized network [2]. Different bit-widths are employed for CNN weight parameters <W> and activation parameters <A>, or, in short, <W:A>. These bit widths are <32:32>, <8:8>, <4:4>, and <2:2>. Lowering bit-width is a form of quantization that offers faster and low-power inference, which is more suitable for low-energy devices. Other than decreasing the bit-width, we create four color modes for each dataset. Besides the original RGB (red, green, blue), we create three different color modes by applying compression methods such as channel reduction and sparsity. The color modes are RGB, grayscale, sparsity on RGB, and sparsity on grayscale. The reason for using four color modes is to evaluate the effectiveness of these color modes in reducing the complexity of image representation, which in turn lowers the computational power. Thus, low-energy devices can process these images because the power requirements are reduced. Finally, we compare and contrast the results.

### 4.1. Model

The ResNet20 model is leveraged, which starts with a conv2d layer and ends with a linear layer with 2 outputs for binary classification. Between these two layers, there are three sequential sub-models. In each of these sub-models, there are three basic blocks. Each basic block contains two pairs of (conv2d, batch normalization) layers, followed by a shortcut link. The quantized model has the same architecture as described above, with the addition of quantization to all conv2d layers except the first layer.

### 4.2. Four Color Modes

**Figure 2:**
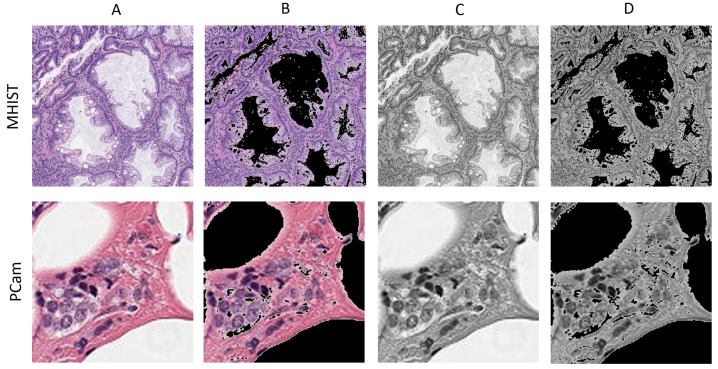
A sample image from the two datasets for each color mode. (**A**) RGB (original), (**B**) sparsity on RGB, (**C**) grayscale, (**D**) sparsity on grayscale.

We consider four different color modes for each dataset. The color modes are RGB, grayscale, sparsity on RGB, and sparsity on grayscale. It is worth mentioning that the intensity of an image pixel varies in a range from 0 to 255, where 0 signifies black and 255 is white. We used python libraries, NumPy and PIL, to create four color modes for each of the two datasets. For grayscale color mode, an original RGB image is converted to grayscale using PIL.Image.convert(L). Then, the number of channels in the image is increased from one to three in order to meet the model input shape requirements. For sparsity on RGB, we examine each pixel in the image. The pixels range from 0 (black) to 255 (white). For any pixel that is 200 or more, the pixel is converted to 0 (black). Thus, we increase an image’s sparsity and the number of zeros. For sparsity on grayscale, the same process of converting pixels of 200 or more to 0 is applied to each grayscale image. Before feeding them to the deep learning model, all images are saved in png format. Figure 2 shows a sample image from each color mode for the two datasets.

### 4.3. Pre-Processing and Training Details

The PCam dataset images are resized from 96×96 to 224×224 to meet the input size of ResNet20. For data augmentation, we apply random horizontal flipping and random vertical flipping. Another pre-processing step is creating the four color modes, which are described in Section 4.2. We trained a ResNet20 model, based on [2]’s implementation, first without quantization, on 32-bit precision. The training is done on PyTorch with a batch size of 32. The training optimizer is Adam, with a momentum of 0.9. The learning rate starts at 0.001 and is changed based on the cosine annealing scheduler [28], and the cross-entropy loss function is applied. The training is performed using PyTorch on a single NVIDIA Tesla V100 GPU [provided by the Holland Computing Center at the University of Nebraska-Lincoln] for 100 epochs, unless otherwise specified. The same training procedure and hyperparameters are used for all four color modes. The second phase of training is done with lower bit-width, as explained in Section 5.1.2. In this training phase, we use the Adam optimizer with a 0.001 learning rate and cosine scheduler.

## 5. Evaluation

In this section, we use three different metrics to evaluate our method, including (i) classification accuracy, (ii) energy usage, and (iii) hardware utilization.

### 5.1. Accuracy Evaluation

The accuracy is defined as the number of correctly classified cases divided by the total number of cases. We calculate the accuracy using sklearn.metrics.accuracy_score and we analyze the accuracy of 32-bit precision without quantization. Then, the accuracy with different quantization configurations is examined. We demonstrate that even with lower bit-width precision, accuracy similar to 32-bit precision can be achieved. Then, we trained it again on a lower bit-width configuration. A lower bit-width of 2, 4, and 8 was chosen for weight <W> and activation <A> parameters, i.e., <W:A> = <2:2>, <4:4>, <8:8>. Hence, we performed for 3 × 2 × 4 = (<W-A> configurations × number of datasets × color modes) = 24 experiments. In Table 3, we report the results of different configurations for each color mode with respect to each dataset.

#### 5.1.1. 32-Bit Precision Results

Here, we show the accuracy results of training and inference on full-precision, 32-bit. Without quantization or lowering the bit-width, the results are as shown in Table 4. It is shown that for the PCam dataset, sparsity on Grayscale color mode produces the best accuracy of 90%. For MHIST, the best accuracy of 76% is acquired by RGB color mode.

#### 5.1.2. Low Bit-Width Precision Results

In this section, we present the results of low bit-width model training. It is worth noting that training of low bit-width models was not performed from scratch, but the training benefited from the knowledge acquired from the 32-bit-width training mentioned in the previous Section 5.1.1. Hence, the status of the models reported in Table 4 is saved and loaded, and training is continued with low bit-width. This pipeline of first training on 32-bit, and then continuing training with lower bit-width, is the pipeline suggested by [2]. The final inference accuracy results with low bit-width training are shown in Table 3. The highest accuracy achieved is 0.77 for RGB and grayscale, 0.73 for sparsity on grayscale, and 0.76 for sparsity on RGB, where the weight parameter bit-width is 4 and activation bit-width is also 4, i.e., bit-width configuration <W:A> = <4:4>. This is for the MHIST dataset. On the other hand, for the PCam dataset, the inference accuracy was 0.85 for RGB on <8:8>. For grayscale color mode, the accuracy achieved 0.89 on <4:4> and <8:8>. For sparsity on grayscale, the accuracy became 0.90 on <8:8>. Finally, for sparsity on RGB, the accuracy was 0.86 on <2:2>.

#### 5.1.3. Comparison to Previous Work

Low bit-width training based on DoReFa-Net was applied to the PCam dataset by [29]. It was shown that with a reduction of 77% of MACs, the error rate is lower by 1% from training on the original RGB color mode compared to training on sparsity on grayscale. Table 5 shows the inference results of ResNet56 when trained on low bit-width based on DoReFa-Net. In Table 5, we compare the best results obtained by [29] to our results for the same configurations. The PCam results shown in Table 5 are as reported in [29]. We performed experiments on MHIST data on the model used in [29]. The results summarized in Table 5 show that, for the PCam dataset, the accuracy increases by a maximum of 0.04 on the <2:2> configuration in the sparsity on grayscale color mode when our model is used. On the other hand, for the MHIST dataset, the accuracy increased by 0.11 for the <8:8> configuration in RGB color mode.

### 5.2. Energy Estimation

**Figure 3:**
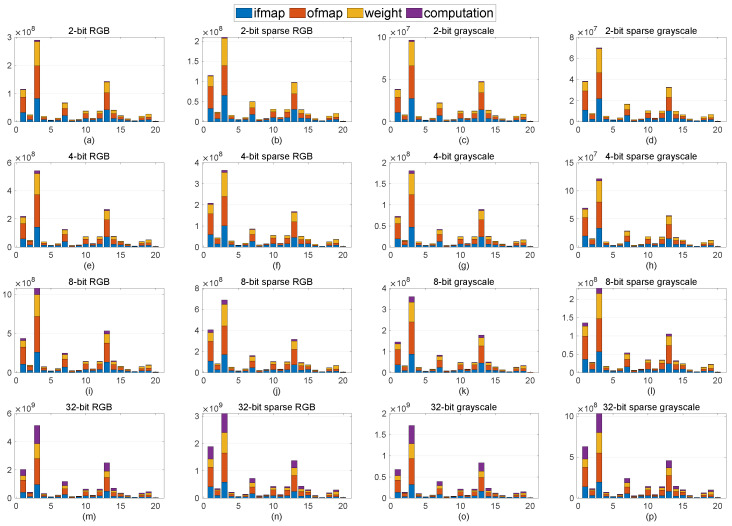
Energy consumption for MHIST dataset, where each row and each column leveraged the same bit-width (e.g., (**a**–**d**)) and identical ResNet20 implementations (e.g., (**a**–**m**)), respectively.

To validate our approach from an energy efficiency perspective, the deep neural network energy estimation tool developed by MIT [30] is utilized. Figure 3 depicts the breakdown of the normalized energy consumption of various implementations of ResNet20, including different bit-widths, sparsity, and color modes, for the MHIST dataset. Each bar consists of communications for input feature map (ifmap), output feature map (ofmap), weight, and total computation. Moreover, from left to right, by applying sparsity or/and different color modes, normalized energy is reduced significantly, whereas, from top to bottom, by increasing the bit-width for an identical color mode, energy consumption is increased. This means that the settings in Figure 3d,m consume the least and most energy, respectively. Although, herein, the first three layers consume considerably higher energy than the other layers, this figure could be varied for different neural network architectures. It is worth noting that, for the PCam dataset, similar behavior is expected as the architecture remains unchanged.

### 5.3. Hardware Utilization

**Figure 4:**
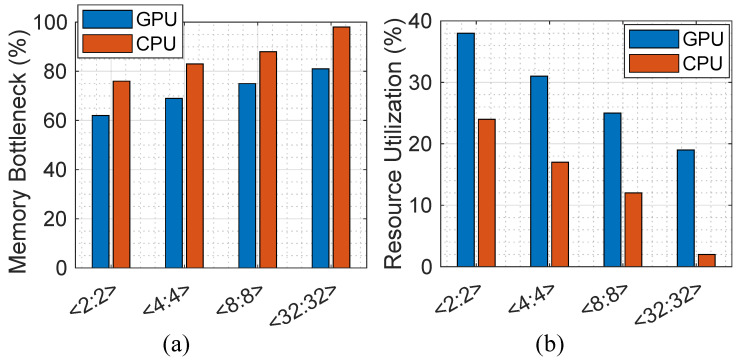
(**a**) The memory bottleneck ratio and (**b**) the resource utilization ratio.

In order to analyze the impact of quantization on hardware implementation, we discuss the memory bottleneck and resource utilization ratio after analyzing the proposed method in both von-Neumann computing platforms, i.e., CPU and GPU. Figure 4a reports the memory bottleneck ratio, defined as the time fraction at which the computation has to wait for data and on-/off-chip data transfer limits the performance (i.e., memory wall happens). The evaluation is performed according to the peak performance and experimentally extracted results for each platform considering the number of memory access times in each bit-width configuration. The results show how the presented quantization method can alleviate the memory wall issue in the modern von-Neumann computer architecture on both CPU and GPU platforms. (1) We observe that the CPU platform imposes a relatively higher memory bottleneck in various <W:A> combinations compared with the GPU. This can be translated into a smaller resource utilization ratio as well. As can be seen in Figure 4b, the GPU implementation with <2:2> configuration can utilize up to 38% of computation resources, though CPU is limited to 24%. (2) The larger the <W:A> bit-widths are, the higher the memory bottleneck ratio is expected to be for both CPU and GPU implementations, reconfirming the importance of the quantization technique.

## 6. Discussion

In this section, we present our experimental results. We first compare the inference results of the 32-bit precision model with various low bit-width models investigated in this paper. Then, we discuss how lowering the bit-width may affect the final classification accuracy results. We use the following bit-width values for weight and activation parameters <W:A>: <32:32>, <8:8>, <4:4>, and <2:2>. As shown in Table 4, for 32 bit-width on MHIST, the highest inference accuracy achieved is 76% in RGB color mode. The other color modes produce lower accuracy when the bit widths are reduced to <2:2>, <4:4>, and <8:8>. The accuracy, as reported in Table 3, is 77% for RGB on MHIST on the <4:4> configuration, which is slightly higher than the full 32-bit-width result. In fact, grayscale color mode reached similar accuracy on <4:4>. This shows that converting RGB images to grayscale may be used to normalize cancerous patterns. Grayscale images can sometimes produce equally accurate or better results than RGB.

On the other hand, for the PCam dataset, Table 4 shows the highest accuracy of 90% for sparsity on grayscale with 32-bit precision. This result is much higher than 84% for the RGB color mode. This confirms our hypothesis that compression methods such as sparsity, and channel reduction such as grayscale, would give comparable or even better results than the original RGB color mode. Moreover, as seen in Table 3, the same accuracy of 90% was achieved when lowering the bit-width to <8:8> with sparsity on grayscale. This proves our hypothesis that a lower bit-width can produce results comparable to the full 32 bit-width.

Finally, the results presented in Table 6 show the detailed classification results of all color modes concerning the two datasets. The true positive rate (TPR) is shown as the sensitivity rate. This rate indicates the number of sick people that are correctly identified as sick. Additionally, the true negative rate (TNR) is presented. This is the specificity rate, which measures the number of healthy people correctly identified as healthy. We have added our baseline method, RGB (original), with a <32:32> configuration that contains no optimization and no quantization. For PCam, the baseline achieved 93% TP on 32 bit-width. The same level of TP is also achievable in the same color mode when lowering the bit-width to <8:8>. Moreover, the same TP is achieved in grayscale with <4:4>. For MHIST, the baseline reached 63% TP on 32 bit-width. However, with a lower bit-width and compressed color mode, a better TP of 77% is achieved.

## 7. Conclusions

In this paper, we have used low-bit-width training of ResNet20 based on [11]. Using a lower bit-width reduces the complexity of the model and therefore uses low power, which is suitable for low-power medical devices. We applied this method to two histopathology datasets: PCam and MHIST. We found that lowering the bit-width, and thus using low power, can achieve results comparable to full-bit-width training of the same model. A specific challenge in the MHIST dataset is the lack of balance in the cases between the two classes. As shown in Table 2, one class represents 69% of the dataset. This makes it difficult to recognize cases of the minority class. There is an inherent limitation in every medical imaging dataset, which is the diversity of the patterns. Some malignant patterns appear similar to benign ones. Both humans and machines have difficulty diagnosing cases due to this. However, humans and machines need to work together to produce a higher-quality diagnosis. Some patterns are more difficult to notice for humans but easier for machines, and vice versa. Thus, machine diagnosis gives another perspective and preliminary recommendation to physicians and pathologists.

In the future, we plan to evaluate the effectiveness of different sparsity thresholds. We used 200 pixels in this paper, as described in Section 4.2. We plan to evaluate 180 and 220 pixels. This should test whether the general pattern is affected if more white pixels are removed. We also plan to implement the model on mobile phones and evaluate the accuracy, energy, and hardware utilization.

## Figures and Tables

**Table 1:** The number of images in PCam dataset and their partitioning for training, validation, and testing. It is clear that this is a balanced dataset.

Class	Train	Val	Test	Total
NonMetastasis (0)	131,072	16,399	16,391	163,862
Metastasis (1)	131072	16369	16,377	163,818
Total	262,144	32,768	32,768	327,680

**Table 2:** The number of images in MHIST dataset and their partitioning into training, validation, and testing. It is clear that this is not a balanced dataset. The HP class covers 69% of the dataset.

Class	Train	Val	Test	Total
HP	1545	155	462	2162
SSA	630	90	270	990
Total	2175	245	732	3152

**Table 3:** Inference accuracy results of training ResNet20 with lower bit-width on weight (W-bits) and activation parameters (A-bits). The result is reported for each dataset with respect to each color mode.

Dataset	Color Mode	W-Bits	A-Bits	Accuracy
PCam	RGB	2	2	0.84
PCam	RGB	4	4	0.84
PCam	RGB	8	8	0.85
PCam	Grayscale	2	2	0.88
PCam	Grayscale	4	4	0.89
PCam	Grayscale	8	8	0.89
PCam	Sp. on Grayscale	2	2	0.88
PCam	Sp. on Grayscale	4	4	0.89
PCam	Sp. on Grayscale	8	8	0.90
PCam	Sp. on RGB	2	2	0.86
PCam	Sp. on RGB	4	4	0.84
PCam	Sp. on RGB	8	8	0.85
MHIST	RGB	2	2	0.74
MHIST	RGB	4	4	0.77
MHIST	RGB	8	8	0.77
MHIST	Grayscale	2	2	0.69
MHIST	Grayscale	4	4	0.77
MHIST	Grayscale	8	8	0.71
MHIST	Sp. on Grayscale	2	2	0.65
MHIST	Sp. on Grayscale	4	4	0.73
MHIST	Sp. on Grayscale	8	8	0.69
MHIST	Sp. on RGB	2	2	0.63
MHIST	Sp. on RGB	4	4	0.76
MHIST	Sp. on RGB	8	8	0.70

**Table 4:** Inference accuracy results of 32-bit-width training and inference on ResNet20.

Dataset	Color Mode	Accuracy
PCam	RGB	0.84
PCam	Grayscale	0.89
PCam	Sparsity on Grayscale	0.90
PCam	Sparsity on RGB	0.83
MHIST	RGB	0.76
MHIST	Grayscale	0.73
MHIST	Sparsity on Grayscale	0.63
MHIST	Sparsity on RGB	0.69

**Table 5:** Comparison of accuracy results with our previous [29].

Dataset	Accuracy (Current)	Accuracy [29]	<W:A>
PCam-rgb	0.84	0.81	<2:2>
PCam-gs	0.88	0.86	<2:2>
PCam-gs-sp	0.88	0.84	<2:2>
PCam-rgb-sp	0.86	0.82	<2:2>
MHIST-rgb	0.77	0.66	<8:8>
MHIST-gs	0.71	0.66	<8:8>
MHIST-gs-sp	0.73	0.66	<4:4>
MHIST-rgb-sp	0.70	0.67	<8:8>

**Table 6:** Detailed results of the best configurations for each color mode: True positive (TP), true negative (TN), false positive (FP), false negative (FN), true positive rate (TPR), true negative rate (TNR), and accuracy. The Activation (A) and Weights (W) bit-width are shown. To have a fair comparison, the baseline method contains no quantization or further optimization. All results are normalized to the predicted value.

Color Mode	TN	TP	FN	FP	TNR	TPR	Acc	<A:W>
PCam:RGB	0.78	**0.93**	0.22	0.07	**0.91**	0.81	0.84	<8:8>
PCam:GS	0.86	**0.93**	0.14	0.07	**0.92**	0.87	0.89	<4:4>
PCam:SP-RGB	0.80	**0.91**	0.20	0.09	**0.90**	0.82	0.85	<8:8>
PCam:SP-GS	0.87	**0.91**	0.13	0.09	**0.91**	0.88	0.89	<8:8>
PCam:RGB [baseline]	0.78	**0.93**	0.22	0.07	**0.92**	0.81	0.84	<32:32>
MHIST:RGB	**0.78**	0.73	0.22	0.27	0.74	0.77	0.77	<8:8>
MHIST:GS	**0.82**	0.68	0.18	0.32	0.72	0.79	0.77	<4:4>
MHIST:SP-RGB	0.75	**0.77**	0.25	0.23	**0.77**	0.76	0.76	<4:4>
MHIST:SP-GS	**0.77**	0.65	0.23	0.35	0.69	0.74	0.73	<4:4>
MHIST:RGB [baseline]	**0.89**	0.63	0.11	0.37	0.71	0.86	0.76	<32:32>

## Data Availability

The data presented in this study are openly available in at https://github.com/basveeling/pcam (accessed on 31 July 2022) and https://bmirds.github.io/MHIST/ (accessed on 31 July 2022).

## Abbreviations

ML	Machine learning
DL	Deep learning
CNN	Convolutional neural networks
RGB	Red, green, blue color mode
SP	Sparsity color mode
GS	Grayscale color mode
<W:A>	<Weight:activation> bit-width configuration
PCam	The PatchCamelyon dataset
MHIST	The Minimalist Histopathology dataset
MACs	Multiply-and-accumulate operation
NumPy	A python library that supports operations on large and multidimensional matrices
PIL	A python image library that supports manipulating and saving images
Ensemble	A group of DL models evaluating one task, e.g., classification
